# Effects of a workplace participatory approach to support working caregivers in balancing work, private life and informal care: a randomized controlled trial

**DOI:** 10.5271/sjweh.4208

**Published:** 2025-05-01

**Authors:** Eline E Vos, Allard J van der Beek, Simone R de Bruin, Karin I Proper

**Affiliations:** 1 National Institute for Public Health and the Environment, Center for Prevention, Lifestyle and Health, Department Behaviour and Health, Antonie van Leeuwenhoeklaan 9, 3721 MA Bilthoven, The Netherlands.; 2 Department of Public and Occupational Health, Vrije Universiteit Amsterdam, Amsterdam Public Health research institute, Amsterdam UMC, 1081 BT Amsterdam, The Netherlands.; 3 Windesheim University of Applied Sciences, Department of Health and Well-being, Research Group Living well with dementia, Campus 2, P.O. Box 10090, 8000 GB Zwolle, The Netherlands.

**Keywords:** caregiving employee, informal caregiver, occupational health professional, RCT, supervisor, supervisor support work–life balance, work–family balance, work–life conflict, work–family conflict, workplace intervention

## Abstract

**Objectives:**

Many employees combine their work with informal care responsibilities for family and friends, potentially impacting their well-being and sustained employability. This study aimed to investigate the effectiveness of a workplace participatory approach (PA) intervention in supporting working caregivers to prevent and solve problems related to balancing work, private life, and informal care tasks.

**Methods:**

We conducted a two-armed randomized controlled trial (ISRCTN15363783) in which working caregivers either received the PA (N=57), under guidance of an occupational professional serving as process facilitator, or usual care (N=59). We recruited 125 working caregivers from four Dutch organizations. Questionnaire-based measurements were assessed at baseline, 4, and 7 months. The primary outcome was work–life imbalance. Secondary outcomes were perceived social support from supervisors and colleagues, role overload, distress and perceived burden of combining work and informal care. Intervention effects were analyzed using intention-to-treat analysis and linear mixed models.

**Results:**

The PA was not effective in reducing work–life imbalance, improving support from colleagues or reducing role overload, distress and perceived burden of combining work and informal care. However, the PA significantly improved perceived social support from supervisors at 4 months [β=0.54, 95% confidence interval (CI) 0.21–0.88] and 7 months (β=0.36, 95% CI 0.02–0.70). Interaction effects indicated that improvement in supervisor support varied depending on the organization.

**Conclusion:**

The PA improved supervisor support but not work–life imbalance. Further research should explore PA effects on working caregivers with and without balance issues and the role of supervisor support in reducing work–life conflict.

In the European Union, one third of people of working age (18–64 years) have caregiving responsibilities, amounting to approximately 100 million individuals (1). With an aging society and rising labor participation rates, the number of workers caring for (aging) relatives or friends in need of care and assistance is expected to increase (2). Caregiving can be a fulfilling but stressful and demanding responsibility and, therefore, have a negative impact on workers' well-being, mental and physical health (3–5). Workers who are struggling to balance their caregiving responsibilities with their work obligations may experience several challenges, such as work and sleep interruptions, insufficient time for relaxation, feelings of stress, exhaustion and loss of focus (6–9). Moreover, they are more likely to be absent due to illness from work, while caregiving can also be an important reason for workers to reduce working hours or leave their job altogether (9–11).

By providing timely support, organizations can assist working caregivers in effectively managing multiple responsibilities and work–life balance, thereby both promoting sustainable employability and continued well-being. To aid in achieving work–life balance, working caregivers have indicated two important workplace needs: (i) social support, which involves being able to discuss and receive understanding for their caregiver role from their employer, supervisors, and colleagues, and (ii) guidance in finding appropriate support and tailored solutions for the specific challenges they face (12–14). The caregiver's supervisor plays a crucial role in support, which may include individual arrangements about tasks and responsibilities, flexible work options, or formal care leave opportunities (12). In this, supervisors have expressed a need for additional knowledge, protocols and resources to assist in meeting their caregiving employees' needs (15, 16).

One potential approach to address working caregivers and supervisors' needs is the participatory approach (PA), a conversation-based stepwise method applied within the occupational setting. The PA has been shown to help workers in tackling various health- and functioning-related challenges (17) and was recently adapted to address workers' issues spanning multiple life domains (eg, financial stress affecting work, health, and personal life) (18). For this study, the PA intervention was further developed for workers with caregiving responsibilities, to help them manage and resolve issues arising from the intersection of work, personal life and caregiving duties. This is done under the guidance of a process facilitator (ie, occupational professional) and with assistance from the employee's supervisor. The aim of this study was to evaluate the effectiveness of the PA intervention in supporting working caregivers to prevent and solve problems related to balancing work, private life, and informal care tasks.

## Program theory

The PA is based on self-determination theory and the positive health approach, emphasizing collaborative stakeholder engagement to address key challenges and promote employees' self-management across physical, emotional, and social dimensions within a supportive environment for change (18). Study outcomes were chosen based on Lindt et al's (2020) Adapted Stress Model (ASM) for caregivers (19), which uses stress and role theories to explain caregiver role conflict and burden. Our primary outcome was the prevention of role conflict (ie, work–care–life imbalance) by engaging PA participants in tailored solution-focused discussions to manage competing demands. We hypothesized that the PA could also positively affect other ASM variables, such as role overload, and social support through collaboration with supervisors and assistance from colleagues. Finally, as the ASM links role stressors to subsequent caregiver stress and burden, these were included as additional outcomes to assess potential broader intervention effects.

## Methods

### Study design, randomization and blinding

We conducted a two-armed (1:1) randomized controlled trial (RCT) in four Dutch organizations: two municipal organizations (~10 000 employees), one governmental organization (~6000 employees) and one university of applied sciences (~3000 employees). Participants were randomly assigned to the intervention group or the usual care control group at the caregiver level within each organization. An independent researcher shared a computer-generated randomization scheme for each organization with the main researcher. Individual participants who had provided informed consent and met the inclusion criteria were added to the randomization schemes, based on the date and time of their completed baseline questionnaire. The researchers and study participants could not be blinded for the group assignment due to the type of intervention; however, group assignments (0 or 1) were blinded during analysis. Process facilitators and supervisors were instructed to only apply the PA to caregivers in the intervention group for the duration of the study (7 months). The study was carried out between May 2022 and May 2024; recruitment ended in October 2023. Facilitators guided multiple PA trajectories at the same time, which were expected to conclude within 4 months. Outcome measurements were assessed at baseline and after 4 and 7 months via questionnaire.

### Recruitment and participants

Participating organizations selected occupational (health) professionals to be trained as process facilitators in the PA, including company social workers (N=9), human resources advisors (N=4), and a sickness absence counsellor (N=1).

Caregiving employees were recruited via internal communication channels (newsletters, emails, blogs, flyers) and through managers encouraging caregivers to join.

Employees were eligible to participate if they had: (i) informal caregiving tasks, regardless of the number of hours of caregiving per week, (ii) ≥20 hours of work/week, and (iii) a labor contract lasting ≥7 months. Informal caregiving was defined as providing voluntary care or support to someone in their social circle with health or functional limitations. Exclusion criteria were: (i) pregnancy/parental leave within 7 months, (ii) on sick leave for >2 consecutive weeks, or (iii) legal conflict with their employer.

### Intervention

**Figure 1:**
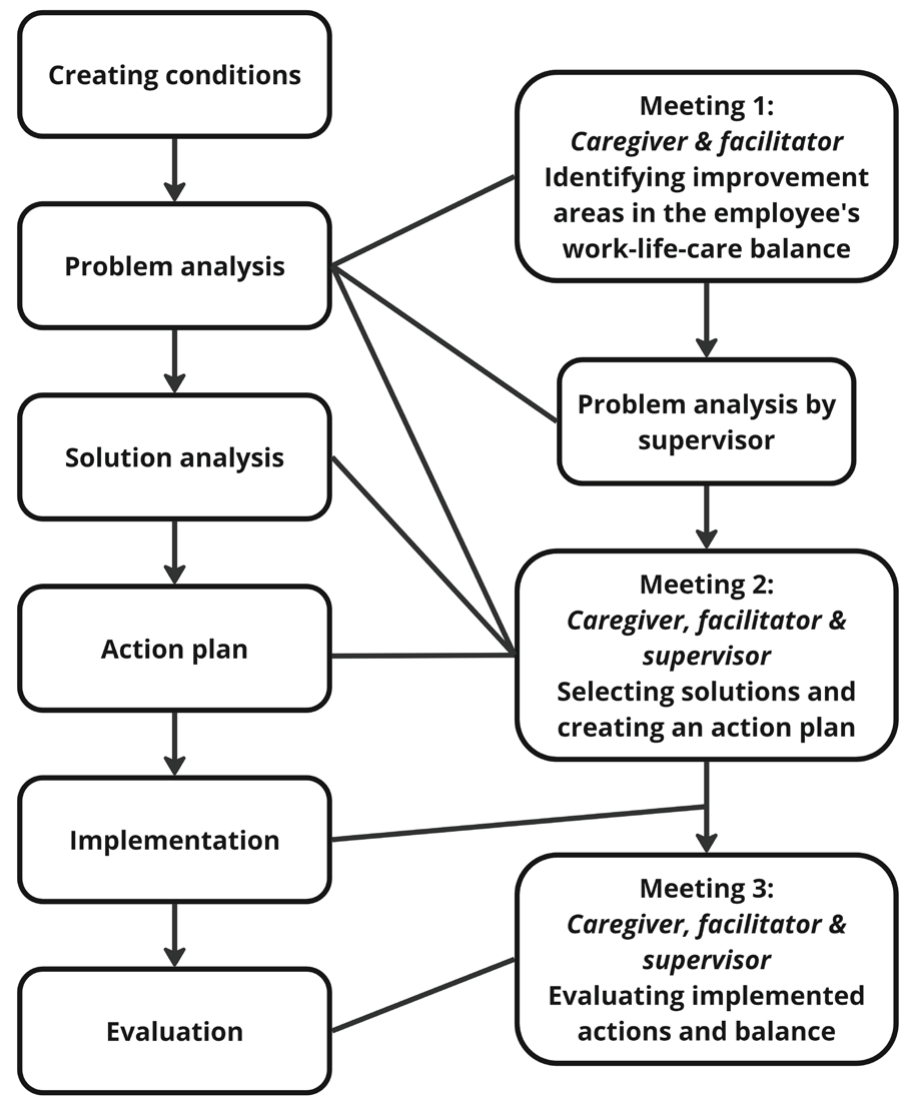
Steps of the participatory approach (PA).

*Workplace PA intervention.* The PA involves a stepwise approach led by a process facilitator who helps caregiving employees – with assistance from their supervisor – to improve the caregiver's work–life–care balance (figure 1). While applied in the work context, the intervention supports caregivers in identifying and implementing changes across the interconnected domains they navigate, including work, caregiving responsibilities, and personal/home life. In the PA methodology, the facilitator's role is focused on process guidance rather than content expertise or therapeutic support. In this, facilitators help ensure active participation and consensus among stakeholders on key problems and solutions. Each participating organization chose for themselves whom to appoint as facilitator. In most cases they chose occupational social workers, who are tasked with supporting employees in work–life balance, while some selected HR advisors who also assist caregivers.

Facilitators attended two 3-hour workshops to prepare for their role. They were encouraged to use the 'Working on Informal Care' toolkit, which includes visual materials and fill-in forms to guide and structure the conversations, intended for collaborative use by all stakeholders during the PA. Training covered the structured steps of the PA, support options for caregivers, and included role-playing exercises to practice guiding caregivers and supervisors through the process.

Facilitators were advised to complete each PA trajectory in three face-to-face meetings over 6–8 weeks, with a first follow-up at 4 months to accommodate guidance of multiple trajectories. The PA begins with a meeting between the facilitator and caregiver to discuss work, private life, and caregiving situations, identifying any problems that may cause imbalance. If few problems are experienced, the focus is on maintaining a good balance and preventing future issues. Meanwhile the supervisor completes a written assignment on potential challenges related to the employee's care situation and a task analysis of their job responsibilities. In the second meeting, the supervisor joins the facilitator and caregiver in prioritizing problems and/or improvements, brainstorming solutions (table 1) and designing an action plan. This action plan includes who is responsible to implement the solutions and in what timeframe. The third meeting evaluates the implementation of actions and their impact on the caregiver's work–life–care balance, with additional actions or monitoring arrangements made as necessary.

**Table 1:** Examples of challenges and solutions addressed in the participatory approach.

Challenges in combining work-life-care	Solutions
Frequent interruptions at work due to receiving calls from the care recipient	Arranging a call schedule with care recipient; informing colleagues about the situation
More time needed to take care of the care recipient in the mornings	Adjusting work schedule to start at 10:00 hours and occasionally work from home
Insufficient time to relax and recuperate	Rearranging work and caregiving tasks so that the caregiver has a weekly free afternoon

### Usual care

Control group participants received usual care and support available to caregiving employees within their organizations. They did not receive the PA during the study period and had no access to the 'Working on Informal Care' toolkit. Both intervention and control group participants could continue to use existing support resources for working caregivers within and outside the organizations.

### Outcome measures

*Primary outcome measures.* The primary outcome was work–life–informal care imbalance, measured using two negative interference scales of the Survey Work-home Interference Nijmegen (SWING) (20). The PA intervention was hypothesized to reduce negative interference between these life domains for working caregivers.

### Negative work/care-to-personal life interference

Negative interference of the combination of work and informal care with the private/home life of working caregivers was measured using an adapted version of the SWING work-to-home interference scale (20). Based on Boezeman et al's study on working caregivers (21), the SWING work-to-home interference scale was modified for this study by replacing the word 'work' with the phrase 'combination of work and informal care'. An example item is: "How often does it occur that you have to cancel appointments with your partner/family/friends, due to the combination of work and informal care?" (see also supplementary table S1: www.sjweh.fi/article/4208). The scale includes 8 items rated on a 4-point scale (never, sometimes, often and always). Scores were summed and averaged, resulting in a score of 0–3, with 3 indicating the highest level of personal life interference.

### Negative informal care-to-work interference

Negative interference of the informal care situation with work life was measured using the negative home-to-work interference scale of the SWING (20). Also following Boezeman et al (21), the SWING home-to-work interference scale was transformed into a care-to-work interference scale by substituting the word 'home' with 'care situation' (supplementary table S1). This 4-item scale measures how often informal care negatively interferes with work life on a 4-point scale (never to always). Scores were summed and averaged from 0–3, with 3 indicating the highest level of care-to-work interference.

### Secondary outcomes

*Perceived social support.* Social support refers to the perceived emotional and instrumental support received from either supervisors or colleagues.

Perceived social support from supervisors was assessed using three questions from the National Monitor Work and Informal Care (22) addressing the ability to discuss one's informal caregiving situation, receive understanding, and obtain support in finding solutions. Perceived social support from colleagues was measured using four questions on the ability to discuss caregiving, delegate tasks, and receive understanding and support in finding solutions. Responses ranged from 1=completely disagree, to 5=completely agree, and the option 'not applicable'. Average scores of 1–5 were calculated, with higher scores indicating greater perceived support. To be included in the average score, ≥2 of 3 items on supervisor support needed to be scored, and ≥3 of 4 items on team support (ie, not filled in as 'not applicable').

### Role overload

Role overload was assessed with an item from the Perceived Burden in Informal Care questionnaire (EDIZ plus) (23): "Combining the responsibility for the person(s) I care for and the responsibility for my work and/or family is not easy". Responses were rated on a 5-point scale (ie, completely agree to completely disagree). For analysis, response categories were reversed, with higher scores indicating more feelings of overload in combining informal care, work and family life.

### Distress

Distress of working caregivers was measured with the 16-item distress scale of the validated Four-dimensional Symptom Questionnaire (4DSQ) (24). Response categories are measured on a 5-point scale (1=no to 5=very often or constantly). After measurement, the distress-scores were aggregated on a 3-point scale (no=0, sometimes=1, all other responses=2), and summated on a scale score of 0–32 (25). A high score on the scale indicates a high level of distress.

### Perceived burden of combining work and informal care

Overall subjective burden of combining work and informal care was assessed using a single question: "All in all, how burdened do you feel by providing informal care in combination with work?" (23). Responses were scored on a 5-point scale (1=not or hardly burdened to 5=overburdened), with higher scores indicating a higher subjective burden.

### Other variables

All covariates and other variables used for the (descriptive) analyses were measured at baseline. Sociodemographic covariates included gender, age, and education level (lower, middle, higher). Lower education was defined as primary school or lower vocational/secondary education, middle as intermediate vocational or higher secondary education, and higher as higher vocational or university education. Long-term health limitations of informal caregivers were assessed by the presence of chronic illnesses, physical or psychological impairments (yes/no). Caregiving characteristics included caregiving hours, number of care recipients, duration of care, and type of caregiving tasks. Work-related characteristics included weekly working hours (including overtime) and autonomy in choosing working hours.

### Sample size

The study's sample size was determined based on the primary outcome 'negative interference between work and home life' from the original SWING questionnaire (20), targeting 80% power at an alpha of 0.05. Using baseline data from Janssen et al (26), an independent statistician calculated necessary sample sizes through extensive simulations. Assuming a moderate effect size (Cohen's d of 0.5) and a correlation of 0.6 between baseline and follow-up values, 500 populations were simulated. Power calculations via ANCOVA tests recommended a total sample size of 100 individuals (50 in each group).

### Statistical analysis

All statistical analyses were performed at employee level, following the intention‐to-treat principle. Statistical analyses were conducted using SPSS 28.0 (IBM SPSS Statistics) and two-tailed P-values <0.05 were considered to be statistically significant. To assess the effectiveness of the PA intervention, linear mixed model analyses were used for both primary and secondary outcomes. In these, the effect at 4 and 7 months were analyzed, respectively, as well as the overall intervention effect. For the two follow-up measurements, an interaction term for time and treatment group was added (27). For all analyses, first, an unadjusted analysis was performed, taking into account the clustering at the level of the subject and the outcome variables' baseline value, after which models were adjusted for predefined confounders age, gender, and education. Effect modification was assessed for organization and for low versus high level of caregiving hours (≤10 versus >10 hours; based on the median found at baseline). Effect modification was considered present if the interaction term's beta-coefficient was P<0.10.

## Results

### Participants

**Figure 2:**
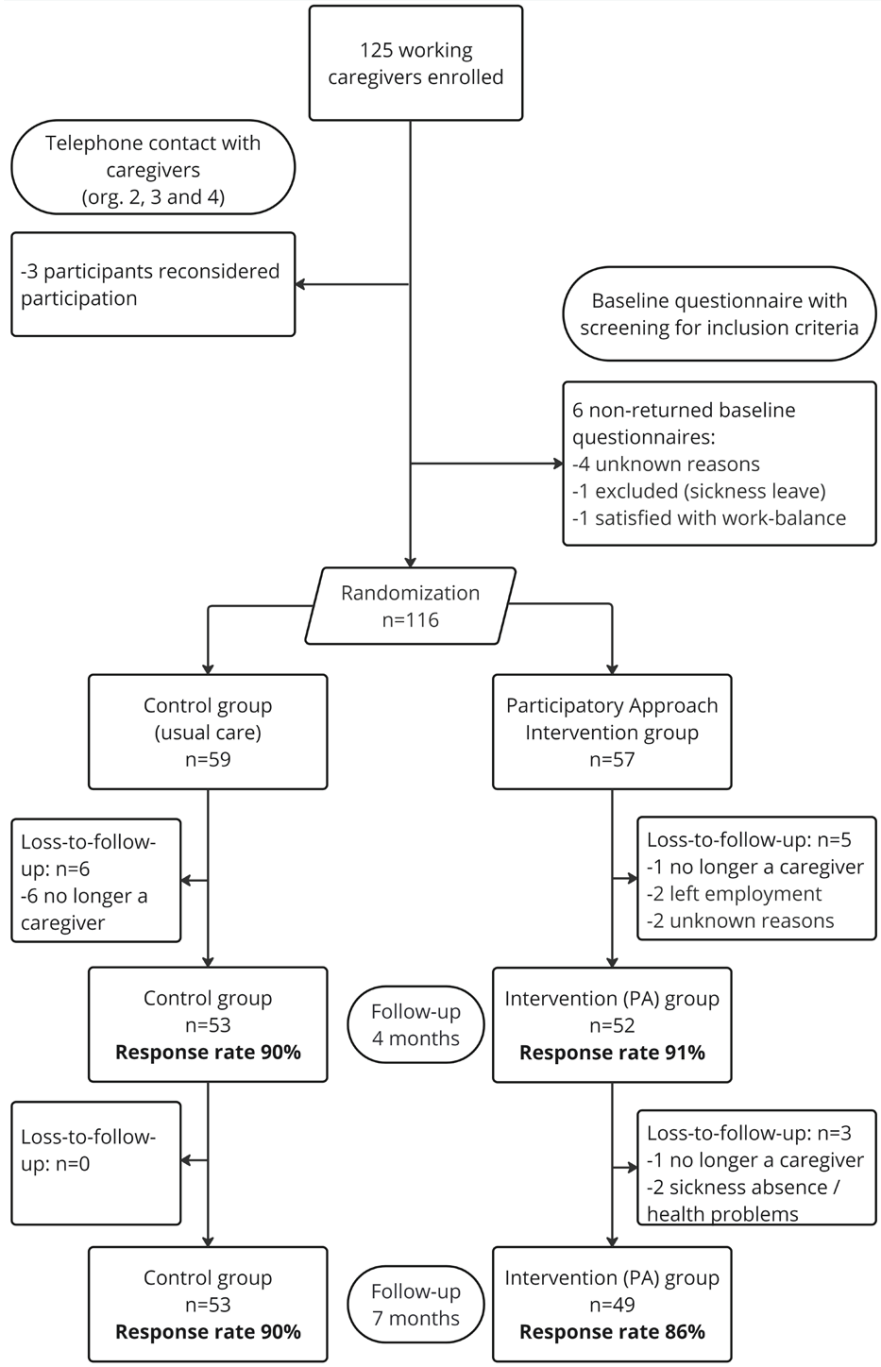
Participant flow chart.

In total, 125 working caregivers within four organizations provided informed consent and enrolled in the study. Of these participants, 9 withdrew or were excluded before randomization (figure 2). In total, 59 participants were allocated to the control group and 57 to the intervention group. In the intervention group, 9 participants did not start the intended (PA) treatment after randomization but were approached for follow-up questionnaires unless they explicitly withdrew from the study in line with the intention-to-treat principle. Seven months after randomization, loss-to-follow up was 10% and 14% in the control and intervention group, respectively. The main reason for loss-to-follow-up was the passing of the care recipient, resulting in participants no longer being informal caregivers.

The majority of participants was female (80%), and highly educated (76%) (table 2). The average age of caregivers were 48.9 and 53.6 years in the intervention and control group, respectively. On average, participants worked 34.5 hours per week, with full-time employment typically defined as 36–40 hours in The Netherlands. A notable proportion of participants (35%) reported long-term health limitations, aligning with literature suggesting that caregivers often face physical and mental health challenges (28). Regarding caregiving responsibilities, a considerable proportion of participants (41%) provided care to multiple individuals, with a median of ten caregiving hours per week. Most participants (90%) had been caregiving for over a year. The most common caregiving tasks included providing emotional support (94%), accompanying medical visits (86%), and organizing or coordinating care (76%), while medical care was the least common task (26%).

**Table 2:** Baseline characteristics of working caregivers enrolled in the study. [SD=standard deviation; IQR=Interquartile range]

			Control group (N=59)				Intervention group (N=57)		
			Mean (SD)	N (%)	Median (IQR)		Mean (SD)	N (%)	Median (IQR)
Age in years			53.6 (7.0)				48.9 (9.7)		
Gender									
	Female			48 (81.4)				45 (78.9)	
	Male			10 (16.9)				12 (21.1)	
	Not specified			1 (1.7)				0 (0.0)	
Educational level									
	Lower			1 (1.7)				4 (7.0)	
	Middle			9 (15.3)				14 (24.6)	
	Higher			49 (83.1)				39 (68.4)	
	Long-term health limitations (yes)			20 (33.9)				21 (36.8)	
Work situation									
	Working hours per week		34.9 (5.8)				34.1 (5.9)		
	Autonomy in choosing work hours								
		Yes, totally		19 (32.2)				20 (35.1)	
		Yes, somewhat		35 (59.3)				32 (56.1)	
		No		5 (8.5)				5 (8.8)	
Informal care situation									
	Number of care recipients (>1)			27 (45.8)				20 (35.1)	
	Number of caregiving hours				10.0 (14.0)				10.0 (25.2)
	Duration of caregiving								
		<12 months		6 (10.2)				6 (10.5)	
		1–5 years		20 (33.9)				22 (38.6)	
		>5 years		33 (55.9)				29 (50.9)	
	Co-living with care recipient (yes)			17 (28.8)				19 (33.3)	
Outcome measures									
	Work/care-to-personal-life interference		1.25 (0.49)				1.23 (0.46)		
	Care-to-work interference		0.71 (0.39)				0.80 (0.46)		
	Social support supervisor		3.71 (1.00)				4.11 (0.81)		
	Social support colleagues		3.63 (0.82)				3.79 (0.89)		
	Role overload		3.81 (0.84)				3.91 (0.91)		
	Distress		12.53 (8.04)				13.95 (9.09)		
	Perceived burden of combining work and informal care		2.59 (0.81)				2.60 (1.03)		

### Intervention effects

The PA had no effect on the primary outcome of work–life imbalance, meaning the negative interference with work life experienced by working caregivers due to their informal care responsibilities, at 4 months (β= –0.14, 95% CI -0.28–0.01), at 7 months (β= –0.07, 95% CI –0.22–0.08), and overall (β= –0.10, 95% CI –0.23–0.03) (Table 3). Similarly, no difference between the control and intervention group was found in negative interference with personal life at 4 months (β=0.01, 95% CI –0.12–0.14), at 7 months (β=0.11, 95% CI –0.02–0.25), and overall (β=0.06, 95% CI –0.05–0.17).

**Table 3:** Intervention effects on primary and secondary outcomes. Adjusted for age, gender and education; crude models not shown as they did not differ from the adjusted models. [CI=confidence interval; WCPI=work/care–personal life interference; CWI=care–work interference].

	T1 (4 months)			T2 (7 months)			Overall effect	
	β ^b^	CI		β ^b^	CI		β ^b^	CI
WCPI (0–3)	-0.14	-0.28–0.01		-0.07	-0.22–0.08		-0.10	-0.23–0.03
CWI (0–3)	0.01	-0.12–0.14		0.11	-0.02–0.25		0.06	-0.05–0.17
Social support supervisor (1–5)	0.54	0.21–0.88		0.36	0.02–0.70		0.45	0.15–0.75
Social support team (1–5)	0.07	-0.23–0.38		0.07	-0.23–0.38		0.07	-0.20–0.34
Role overload (1–5)	-0.21	-0.53–0.12		-0.08	-0.41–0.25		-0.15	-0.43–0.14
Distress (0–32)	-0.61	-3.44–2.21		0.42	-2.43–3.28		-0.10	-2.48–2.27
Perceived burden of combining work and informal care (1–5)	-0.08	-0.35–0.19		0.12	-0.16–0.39		0.01	-0.23–0.25

^b^ A negative beta (β) indicates less interference of life domains, less role overload, less experienced distress, and less perceived burden in the intervention group compared to the control group. A positive beta (β) indicates more interference of life domains, more perceived burden, more experienced distress, and higher social support from supervisor and colleagues in the intervention group compared to the control group.

Regarding secondary outcomes, the intervention demonstrated a statistically significantly positive effect on perceived social support from supervisors, at 4 months (β=0.54, 95% CI 0.21–0.88), at 7 months (β=0.36, 95% CI 0.02–0.70), and overall (β=0.45, 95% CI 0.15–0.75) (Table 3). Analyses revealed an interaction effect with organization, indicating that the observed effects on supervisor support varied across different organizations. Specifically, the effect of the intervention on supervisor support was only found to be statistically significant for organization 2 (β=1.24, 95% CI 0.73–1.76).

No statistically significant differences between control and intervention group were found for any of the secondary outcomes on support from colleagues, distress, role overload and perceived burden of combining work and informal care. Furthermore, no significant interaction effects with organization or high versus low number of caregiving hours were found for these outcomes.

## Discussion

The workplace PA was not effective in reducing work–life imbalance, as measured by interference in both work and personal life domains for working caregivers with varying burden levels. The PA was also not effective in reducing role overload, distress and perceived burden of combining work and informal care or in improving social support from colleagues. However, the PA improved perceived social support from the supervisor.

Our study is among the first RCT to investigate a workplace intervention targeting work–life balance for employed caregivers (29). While applied within the workplace, the intervention took a preventive and integrated approach by addressing challenges across work, care, and personal life . This aligns with research showing that caregivers often face interconnected issues across these domains and can benefit from multi-domain support, including discussing work-care balance and receiving workplace support (12). Further studies (30, 31) have shown that challenges in one life domain often impact well-being in others, underscoring the need for integrated interventions.

Our finding that the PA increased social support from supervisors but did not significantly improve work–life balance outcomes, such as interference, or more distal outcomes, such as caregiver stress and burden, aligns with another PA intervention study on workers with rheumatoid arthritis (32). In that study, supervisor support improved, whereas the distal outcome of work functioning did not. Based on our conceptual model (19), we hypothesized that the PA would reduce work–life imbalance directly and indirectly by first increasing supervisor support. Various studies suggest social support can exert both direct and buffering effects on work–life balance (33–36). For instance, supervisor support and related access to resources have been shown to protect working caregivers against depressive symptoms (37). However, research has indicated delayed effects of perceived supervisor support on health and work outcomes, and these time-lagged effects have been understudied (38). If such lagged effects exist, the short follow-up period of our study might have limited our ability to observe positive effects of supervisor support on work–life balance. We would, however, expect to see some improvement in work–life balance at the 7- compared to 4-month mark, which was not the case. A more likely explanation may lie in a related process evaluation to this RCT (39), revealing that caregivers did not always involve their supervisor in the PA, particularly when already satisfied with their work–life balance and support. Supervisor support significantly improved only in the organization where nearly all caregivers actively engaged their supervisors. This indicates that the PA's benefits on supervisor support can be substantial when supervisors are effectively involved, but the general lack of supervisor involvement may have limited subsequent positive outcomes on work–life balance. Another complicating factor in disentangling the effects of supervisor support on work–life balance is that the SWING questionnaire used to measure work–life imbalance lacked a specific recall period (20). It is possible that supervisor support increased, but its potential effects are not reflected in the results because participants may have been recalling experiences over too long a period. Thus, further research is needed to explore the role of supervisor support in ultimately mitigating work–life conflict over time.

Apart from the role of supervisor support, the PA's lack of effect on work–life balance might be explained by the limited implementation of actions aimed at improving it. Our process evaluation showed that while most caregivers completed the first step of assessing challenges in their work–life–care situation (48 out of 57), only 29 implemented actions, averaging three actions each (39). Again, this limited uptake may be attributed partly to caregivers' existing satisfaction with their balance. Additionally, nearly all participants had some autonomy in choosing their schedules, limiting flexibility as an avenue of action. For those caregivers who did face challenges, the actions implemented during the 3-meeting PA may not have been (impactful) enough to improve work–life balance. Relatedly, in the questionnaire caregivers frequently cited significant life changes, such as health issues, relocations, care recipient transitions to nursing homes, and bereavement, which affected their work–life balance. Previous research has identified work arrangements as important for alleviating work–care conflict, but adjustments to the care situation are at times also necessary (40). However, important determinants of perceived balance, such as caregiving load and care recipient dependency (40), are not always modifiable or easily addressed through interventions in the workplace context. This suggests that further support in the care context may be necessary to adequately address work–life balance for some caregivers.

Finally, the SWING questionnaire defined work–life imbalance as 'interference', which not all participants experienced. The intervention targeted caregivers with varying burden levels to prevent new issues and address existing ones. In particular, the primary outcome 'work interference' had low baseline levels (averaging 0.75 on a scale of 3), making improvement more challenging due to the floor effect (41). However, this does not discount the potential benefits of a preventive PA intervention for working caregivers who have not yet experienced imbalance, as early discussion may prevent more severe or burdening situations later. As noted earlier (42), demonstrating prevention necessitates extensive follow-up periods, which were not feasible in this study. Alternatively, future research could explore PA effects among subgroups of working caregivers with and without existing work–life balance issues and health challenges. This approach could help differentiate between preventive and treatment effects, offering valuable insights into which caregivers may benefit most from the PA (43).

### Strengths and limitations

This study has several strengths and limitations. A key strength is the RCT design and its statistical power. Additionally, the study maintained a low loss to follow-up post-randomization, except when individuals ceased to be caregivers. However, the study population is not representative of the general population of working caregivers, as it had limited participation from male caregivers, those with lower educational levels, and those with less autonomy in choosing their schedules. This is likely due to the fact that the participating organizations consisted of predominantly higher-educated workers and those with more flexible working arrangements, leading to an overrepresentation of these groups in the sample. In work environments where such options for flexibility are unavailable, it may be more challenging to improve work–life balance using the PA. Another limitation is the lack of heterogeneity in recruited and participating organizations. For example, there were no commercial organizations or small and medium enterprises, where we might expect different effects of the PA due to varying work environments, cultures, and support options. Furthermore, while supervisors may have been responsible for employees in both the intervention and control groups, they were instructed to apply the PA only to intervention group employees. As supervisors had no PA involvement with control group caregivers, their names were not collected for these cases. Given the PA's focus on personal challenges and tailored solutions, we believe any potential contamination through supervisor behavior is likely minimal and unlikely to have influenced the study's results. Additionally, the SWING questionnaire's lack of a recall period may have affected the results, as participants could have been recalling experiences over an extended period, including before the intervention. Lastly, the relatively short follow-up duration might have limited the ability to capture the full benefits of the preventive approach, particularly since the benefits of early discussions on work–life balance may take longer to manifest.

### Concluding remarks

This RCT demonstrated that a workplace PA effectively enhanced perceived supervisor support for working caregivers. However, the PA had no other positive effects, neither on the primary outcome of work–life imbalance nor on role overload, social support from colleagues, distress, or the perceived burden of combining work and informal care. Given that supervisor support is an important predictor of various health and work-related outcomes, including work–life balance, future research should investigate the role of supervisor support in the PA's effectiveness on work–life balance. Furthermore, future research should explore PA intervention effects among working caregivers with and without balance issues, and also what is needed to maintain healthy work–life balance outcomes.

## Supplementary material

Supplementary file 1
