# Supplementary material for: Effects of a workplace participatory approach to support working caregivers in balancing work, private life and informal care: a randomized controlled trial
Source: Scand J Work Environ Health. 2025 Apr 27;51(3):181–90. doi: 10.5271/sjweh.4208 (PMC12062804; doi:10.5271/sjweh.4208)
Supplement: Supplementary file 1 [file SJWEH-51-181-S001.pdf]

Effects of a workplace participatory approach to support working caregivers in balancing work, private life and informal care: a randomized controlled trial[1]

by Eline E Vos, MSc[2] Allard J van der Beek, PhD,[2] Simone R de Bruin, PhD,[3] Karin I Proper, PhD [1, 2]

1. Supplementary material
2. Correspondence to: Eline E. Vos, National Institute for Public Health and the Environment, Center for Prevention, Lifestyle and Health, Department Behaviour and Health, Antonie van Leeuwenhoeklaan 9, 3721 MA Bilthoven, The Netherlands. [E-mail: eline.vos.02@rivm.nl]

Table S1. Measured items in primary outcomes Work/care to personal life interference (WCPI) and Care to work interference (CWI). Answer categories: Never, Sometimes, Often, Always.

Item	WCPI	CWI
1.	How often does it occur that you become irritable with your partner/friends/family because the combination of work and informal care is demanding?	How often does it occur that you had difficulty concentrating on your work because you were worried about matters in your caregiving situation?
2.	How often does it occur that you have difficulty meeting your obligations at home because you are constantly thinking about your work, in combination with your caregiving responsibilities?	How often does it occur that your caregiving situation caused irritations that you took out on your colleagues at work?
3.	How often does it occur that you have to cancel appointments with your partner/family/friends, due to the combination of work and informal care?	How often does it occur that your work performance declined due to problems with the person/people you care for?
4.	How often does it occur that the time you spend on work and informal care makes it difficult for you to meet your obligations at home?	How often does it occur that you did not feel like going to work because of problems with the person/people you care for?
5.	How often does it occur that, due to the combination of work and informal care, you have no energy left to do enjoyable things with your partner/family/friends?	
6.	How often does it occur that you have so much to do because of the combination of work and informal care that you do not have time for your hobbies?	
7.	How often does it occur that the demands of combining work and informal care make it difficult for you to feel relaxed at home?	
8.	How often does it occur that the combination of work and informal care takes up time you would rather spend with your partner/family/friends?	
